# miR-96-5p targets PTEN to mediate sunitinib resistance in clear cell renal cell carcinoma

**DOI:** 10.1038/s41598-022-07468-x

**Published:** 2022-03-03

**Authors:** Sang Eun Park, Wonju Kim, Ji-Ye Hong, Dayeon Kang, Seulki Park, Jungyo Suh, Dalsan You, Yun-Yong Park, Nayoung Suh, Jung Jin Hwang, Choung-Soo Kim

**Affiliations:** 1 Asan Institute for Life Sciences, Asan Medical Center, 88, Olympic-ro 43-gil, Songpa-gu, Seoul, 05505 Republic of Korea; 2 Department of Pharmaceutical Engineering, College of Medical Sciences, Soon Chun Hyang University, 22, Soonchunhyang-ro, Shinchang, Asan, Chungnam 31538 Republic of Korea; 3 Department of Medical Sciences, General Graduate School, Soon Chun Hyang University, Asan, Chungnam 31538 Republic of Korea; 4 Disease Target Structure Research Center, Korea Research Institute of Bioscience and Biotechnology (KRIBB), Daejeon, 34141 Republic of Korea; 5 Department of Urology, University of Ulsan College of Medicine, Asan Medical Center, 88, Olympic-ro 43-gil, Songpa-Gu, Seoul, 05505 Republic of Korea; 6 Department of Life Science, Chung-Ang University, Seoul, 06911 Republic of Korea

**Keywords:** Cancer, Urology

## Abstract

A multiple receptor tyrosine kinase inhibitor, sunitinib, is a first-line therapy for clear cell renal cell carcinoma (CCRCC). Unfortunately, it has the major challenges of low initial response rate and resistance after about one year of treatment. Here we evaluated a microRNA (miRNA) and its target responsible for sunitinib resistance. Using miRNA profiling, we identified miR-96-5p upregulation in tumors from sunitinib-resistant CCRCC patients. By bioinformatic analysis, PTEN was selected as a potential target of miR-96-5p, which showed low levels in tumors from sunitinib-resistant CCRCC patients. Furthermore, *PTEN* and miR-96-5p levels were negatively correlated in a large The Cancer Genome Atlas kidney renal clear cell carcinoma cohort and high miR-96 and low *PTEN* represented poor prognosis in this cohort. Additionally, four-week sunitinib treatment increased miR-96-5p and decreased PTEN only in tumors from a sunitinib-resistant patient-derived xenograft model. We found a novel miR-96-5p binding site in the *PTEN* 3′ UTR and confirmed direct repression by luciferase reporter assay. Furthermore, we demonstrated that repression of PTEN by miR-96-5p increased cell proliferation and migration in sunitinib-treated cell lines. These results highlight the direct suppression of PTEN by miR-96-5p and that high miR-96-5p and low PTEN are partially responsible for sunitinib resistance and poor prognosis in CCRCC.

## Introduction

Worldwide, approximately 338,000 new cases of renal cell carcinoma (RCC) are diagnosed and 175,000 patients die from RCC each year^1^. Clear cell renal cell carcinoma (CCRCC) is the most common subtype of this disease, representing 75–80% of RCC cases, which is associated with the loss of the von Hippel–Lindau (VHL) gene^2^. VHL loss is associated with the accumulation of hypoxia-inducible factor 1 (HIF-1) and increased transcription of vascular endothelial growth factor (VEGF) and platelet-derived growth factor (PDGF), leading to intense vascularity^2^. For this reason, therapeutic agents targeting VEGF receptor (VEGFR) signaling have been shown to improve disease control. One of these drugs is sunitinib, which inhibits multiple kinase receptors including VEGFR, PDGF receptor (PDGFR), FMS-like tyrosine kinase 3 (FLT-3), cKIT, and rearranged during transfection (RET)^2^. Its inhibitory actions play roles in both tumor angiogenesis and cancer cell proliferation. CCRCC is generally resistant to chemotherapy or radiation and responds poorly to cytokines including interferon α and interleukin-2. Although treatment with sunitinib showed slightly longer overall survival (OS) than cytokine therapies, the initial response rate is 30%–40% and disease progression occurred in sensitive patients who survived 6–15 months after sunitinib treatment^2^. This indicates that there is de novo and acquired resistance against sunitinib in CCRCC.

MicroRNAs (miRNAs) are endogenous small noncoding RNAs (~ 22 nucleic acids) that control gene expression by mRNA cleavage or halting translation via binding to the complementary sequence of 3′ untranslated region (3′ UTR) of target genes. Since miRNAs play important regulatory roles in gene expression under normal conditions, the dysregulation of miRNAs is frequently observed in many types of cancer, acting as a tumor suppressor or contributing to tumorigenesis^3^. Many researchers have suggested that monitoring the expression of miRNAs in tumor tissues and liquid biopsies from cancer patients may be effective for accurately determining the diagnosis, prognosis, and drug sensitivity^4^. In accordance with this trend, substantial information on CCRCC has been accumulated. The Cancer Genome Atlas (TCGA) Research Network has published comprehensive information on the molecular characterization of CCRCC, including miRNA profiling, and demonstrated a strong association of miR-21 with a worse outcome in CCRCC^5^. Independent groups reported the association of sunitinib resistance with a decrease in miR-141 or miR-101 and an increase in miR-942 or -452-5p in CCRCC patients^6–9^. However, the mechanisms underlying sunitinib resistance and potential predictive biomarkers reflecting drug responsiveness in a clinical context have remained unclear. A deeper understanding of the complex molecular regulatory mechanisms might be beneficial to discover biomarkers for the selection of sensitive or acquired resistance against sunitinib in CCRCC patients.

The aim of this study is to identify the miRNAs in tumors that might predict sensitivity to sunitinib and its target mRNA in CCRCC patients. We performed miRNA array analysis in tumors and normal tissues from sunitinib-sensitive and -resistant patients and selected miR-96-5p, which is upregulated in sunitinib-resistant tumors compared with the level in sunitinib-sensitive tumors. Using target prediction and pathway analyses, we predicted *PTEN* as an mRNA target of miR-96-5p and demonstrated the direct regulation between them by luciferase reporter assay and the introduction of miR-96-5p mimic or inhibitor in CCRCC cell lines. High levels of miR-96 and low levels of *PTEN* are associated with a poor prognosis in the kidney renal clear cell carcinoma (KIRC) cohort of TCGA. In addition, the expression of miR-96-5p was downregulated in a sunitinib-resistant patient-derived xenograft (PDX) animal model, compared with the level in sensitive PDX. These results suggest that miR-96-5p mediates sunitinib resistance in CCRCC in a PTEN-dependent manner.

## Results

### Identification of differentially expressed miRNAs in tumors from CCRCC resistant to sunitinib treatment

**Figure 1:**
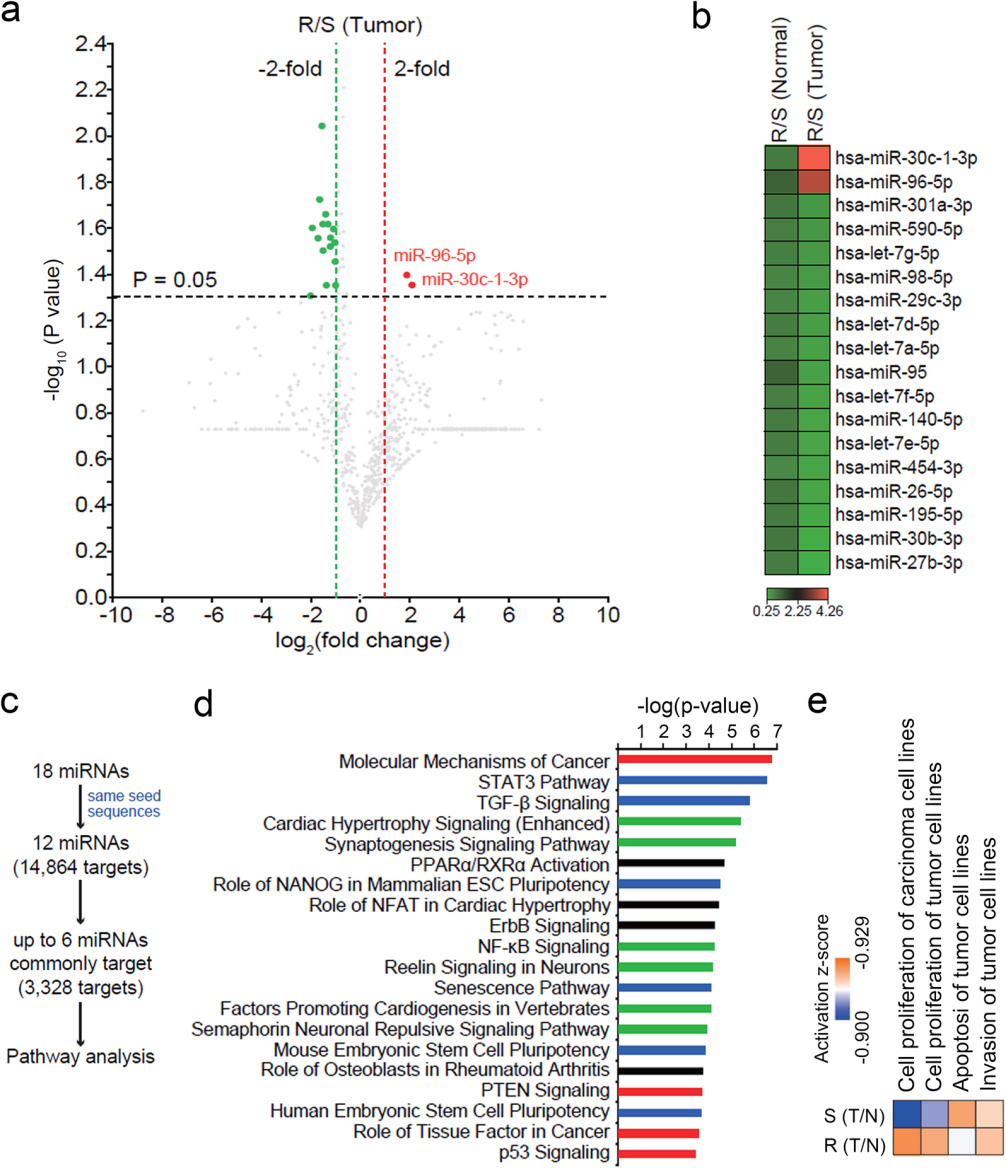
Upregulation of miR-96-5p in tumors from sunitinib-resistant clear cell renal cell carcinoma (CCRCC) patients. (**a**) Volcano plot of differentially expressed miRNAs from microarray of sunitinib-resistant and sunitinib-sensitive CCRCC patients. (**b**) Heatmap of 18 miRNAs differentially expressed in resistant samples compared with sensitive samples in tumors. (**c**) Schematics of target prediction and pathway analysis with differentially expressed miRNAs presented in (a) and (b). (**d**) Predicted common targets of differentially expressed miRNAs were subjected to Ingenuity Pathway Analysis (IPA), and the top 20 enriched canonical pathways are shown (red bars: pathways directly correlated with cancer; blue bars: pathways of organismal growth and development; green bars: pathways of cellular growth, proliferation, and development). (**e**) A heatmap of the cellular and biofunctions enriched in resistant samples compared with sensitive samples. Orange (positive z-score) or blue (negative z-score) color codes represent the activation or inhibition of the given cellular functions.

We performed global miRNA profiling using frozen tissues from CCRCC tumors and adjacent noncancerous kidney tissues of six patients who received sunitinib treatment. The clinical characteristics are summarized in Table 1. The mean progression-free survival (PFS) and OS of the resistant group were 4.7 $\pm$ 3.1 and 7.3 $\pm$ 5.5 months, respectively. The corresponding values in the sensitive group were 41.3 $\pm$ 9.3 and 83.3 $\pm$ 18.2 months, respectively. The mean number of metastatic sites was the same at 1.3 $\pm$ 0.6 in the two groups and the mean ages were similar, 54.7 $\pm$ 13.7 and 56.3 $\pm$ 6.5, for resistant and sensitive patients, respectively. Among these samples, eleven samples (2 normal sensitive, 3 normal resistant, 3 tumor sensitive, and 3 tumor-resistant samples) passed the RNA quality check and could be used for microarray analysis. After data normalization, 345 miRNAs showed raw signal intensity over 10 in at least 6 of 11 samples and classified as reliable genes. The miRNA profile of normal and tumor samples clustered together regardless of sunitinib sensitivity (Supplementary Fig. S1 and Supplementary Table S1). Because we were interested to find miRNAs responsible for drug resistance, we then focused on tumor samples. Within the tumor biopsies, 18 miRNAs (2 upregulated and 16 downregulated) were differentially expressed in resistant samples compared with the levels in sensitive samples (*P* < 0.05) (Fig. 1a). These differences were clearly observed only in tumors, but not in normal tissues (Fig. 1b).

**Table 1:** Clinical characteristics of 6 CCRCC patients.

Characteristic		Samples	
		Resistant* (n = 3)	Sensitive** (n = 3)
Sex	Male	3	2
	Female	0	1
Age	Mean	54.7	56.3
	std	13.7	6.5
# of metastatic site	Mean	1.3	1.3
	std	0.6	0.6
PFS (month)	Mean	4.7	41.3
	std	3.1	9.3
OS (month)	Mean	7.3	83.3
	std	5.5	18.2
5 year mortality	Death	3	0
	Survival	0	3
Duration of sunitinib response (cycle)	Mean	3.7	24.3
	std	2.1	7.4

*PFS* progression-free survival, *OS* overall survival.

*Resistant: sunitinib-resistant CCRCC patients.

**Sensitive: sunitinib-sensitive CCRCC patients.

To understand the regulatory mechanisms of miRNAs differentially expressed in resistant tumor samples, we set out to determine their mRNA targets. Because two miRNAs (miR-301a-3p and miR-454-3p) and also six miRNAs (let-7a-5p, let-7d-5p, let-7e-5p, let-7f-5p, let-7g-5p, and miR-98-5p) showed the same seed sequences, we determined the potential target genes for 12 miRNAs using TargetScan (release 7.2). Initial screening identified 14,864 target genes. Among those, 3,328 putative targets were predicted to be regulated by up to 50% of the differentially expressed miRNAs. The function and network analyses were then performed on these 3,328 genes using the Ingenuity Pathway Analysis (IPA) software (Fig. 1c). The top 20 canonical pathways enriched in the target genes were predominantly associated with cancer pathophysiology (Fig. 1d). For example, four pathways, namely, molecular mechanisms of cancer, PTEN signaling, role of tissue factor in cancer, and p53 signaling, were directly correlated with cancer (Fig. 1d, red bars). Intriguingly, five pathways, such as the STAT3 pathway, were categorized into organismal growth and development (Fig. 1d, blue bars) and six pathways, including cardiac hypertrophy signaling, were involved in cellular growth, proliferation, and development (Fig. 1d, green bars). Furthermore, the cellular and biofunctions including cell proliferation and invasion of tumor cell lines were significantly upregulated in tumor-resistant groups compared with the levels in sensitive groups (Fig. 1e, activation z-score from − 0.900 to 0.929). In contrast, the apoptosis of tumor cell lines was predicted to be downregulated in in tumor-resistant groups (Fig. 1e). Taking these findings together, we found that 18 miRNAs were differentially expressed in sunitinib-resistant tumors from CCRCC and that their predicted target genes were highly involved in cancer metabolism and cellular growth and development.

### High miR-96 and low *PTEN* are associated with poor prognosis and sunitinib resistance in CCRCC

**Figure 2:**
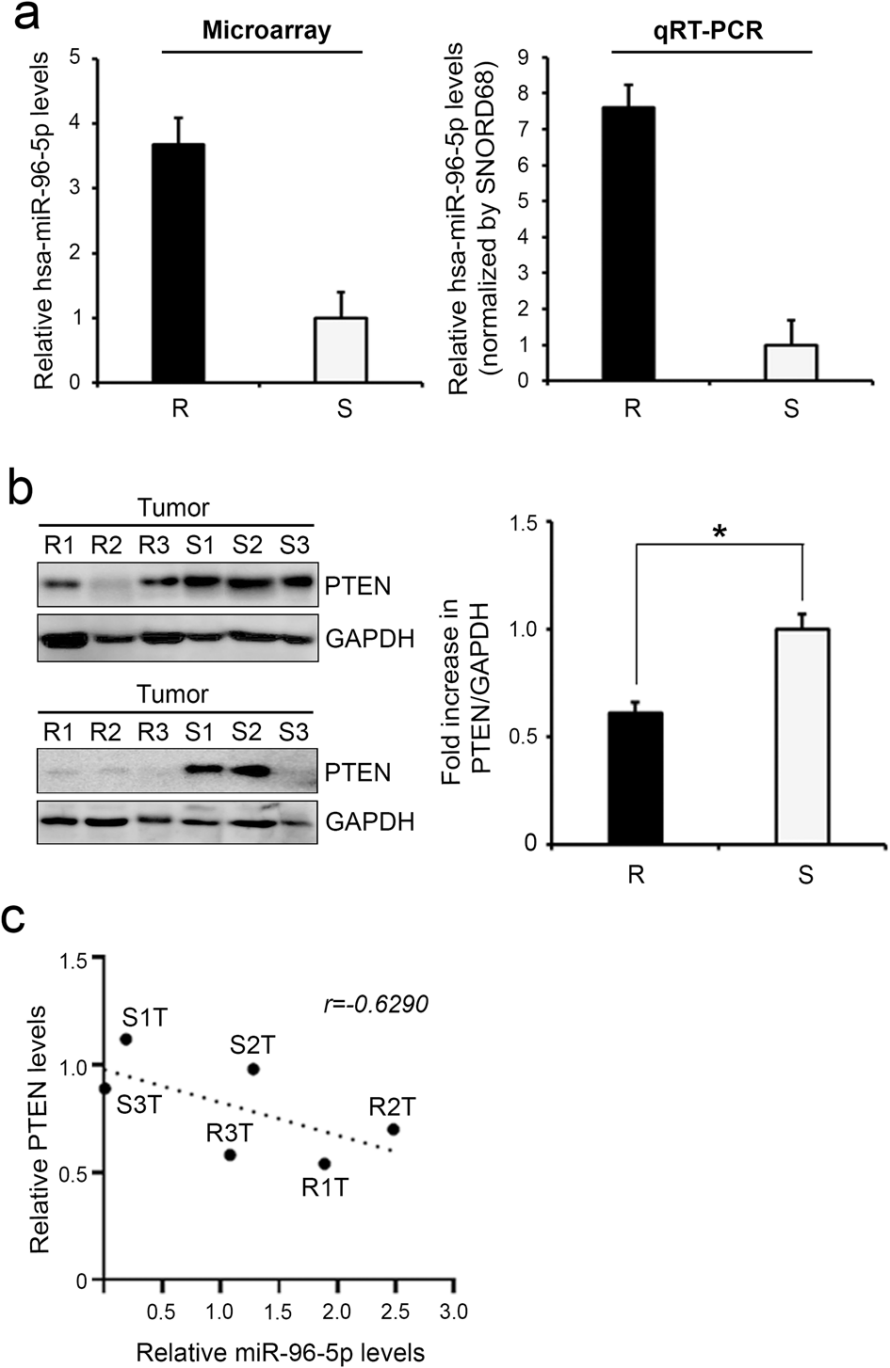
Expression levels of miR-96-5p and PTEN in patient samples. (**a**) The fold changes detected by microarray are denoted in a bar graph. Real-time RT-PCR validation of the miR-96-5p expression levels in the same patient samples used in the microarray. (**b**) Western blot (left) and quantification (right) of PTEN proteins in tumor tissues from sunitinib-resistant (R1, R2, and R3) and sunitinib-sensitive (S1, S2, and S3) CCRCC patients. Samples were collected from two different regions of tumors. GAPDH was used as a loading control. Data are presented as mean ± s.e.m. (n = 3). Significant results are presented as *P < 0.01. (**c**) The levels of miR-96-5p determined by qRT-PCR in fresh frozen tissue were compared with the corresponding PTEN proteins determined by Western blotting.

Among the two upregulated miRNAs in sunitinib-resistant tumors, we focused on miR-96-5p, which is known as an oncogenic miRNA in multiple cancers including prostate, ovarian, endometrial, cervical, breast, colon, lung, and head and neck cancer^10–15^. The level of miR-96-5p was upregulated 3.7-fold in miRNA microarray in tumors of poor responders compared with that for sensitive tumors (Fig. 2a, left). Consistent with this, when we confirmed the expression levels by quantitative real-time PCR (qRT-PCR), the expression of miR-96-5p showed a 7.6-fold increase in tumors of poor responders compared with that of sensitive tumors (Fig. 2a, right). We next determined the target of miR-96-5p to understand the molecular basis of the high levels of miR-96-5p in tumors of poor responders. TargetScan predicted 4,131 transcripts to be potentially targeted by miR-96-5p. Considering its well-known functions as a tumor suppressor in the regulation of cell survival, migration, proliferation, and metabolism, we selected PTEN as a potential target of miR-96-5p and tested its possible role in controlling resistance to sunitinib in cancer. To check the possibility that miR-96-5p modulates PTEN expression, we first determined the levels of PTEN protein in tumors collected from two different regions by Western blotting. The levels of PTEN protein were considerably low in sunitinib-resistant tumors compared with those in sensitive tumors (Fig. 2b), which showed the inverse correlation with miR-96-5p (Fig, 2c). This negative correlation suggests that PTEN might be regulated by miR-96-5p, the level of which is increased in sunitinib-resistant CCRCC.

**Figure 3:**
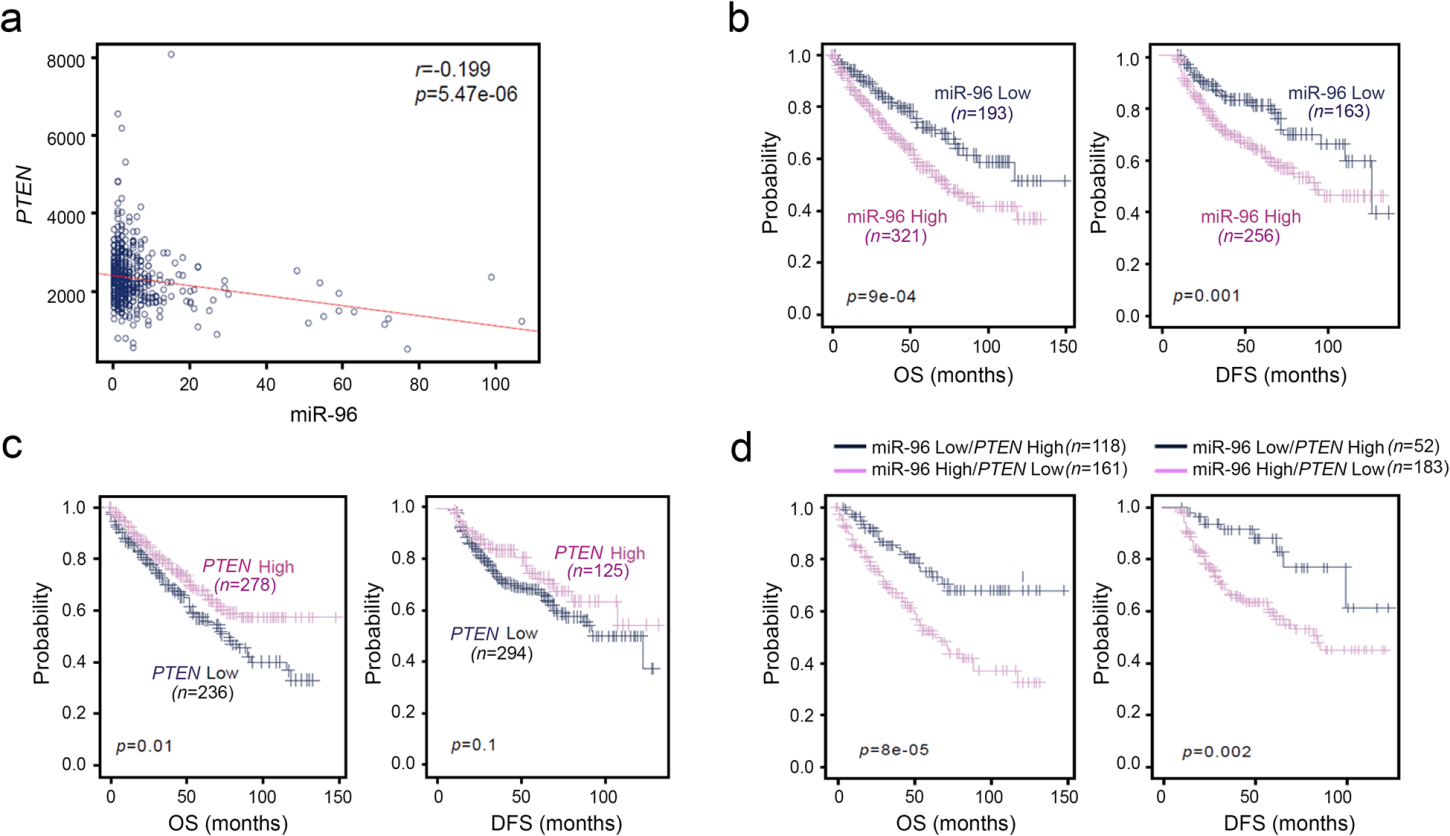
Associations of higher miR-96 and lower *PTEN* expression levels with a poor prognosis in CCRCC patients. (**a**) The correlation between miR-96 and *PTEN* expression in TCGA CCRCC patients (KIRC) was estimated using Pearson’s correlation test. (**b–d**) Kaplan–Meier curves of overall survival (OS) and progression-free survival (PFS) for miR-96 and *PTEN* expression in CCRCC patients. miR-96 and *PTEN* expression was dichotomized into high- and low-expression categories (b, c). CCRCC patients in KIRC were dichotomized based on the expression of miR-96 and *PTEN* and patients with higher expression levels of both miR-96 and *PTEN* or lower expression levels of both miR-96 and *PTEN* were identified for analysis (**d**).

We investigated this possibility using publicly available patient data. First, we analyzed whether the PTEN levels are inversely correlated with miR-96 expression in a large cohort, in the TCGA KIRC data set composed of 514 patients. The level of *PTEN* mRNA was negatively associated with miR-96 (*r* =  − 0.199) in the KIRC cohort, as shown in a scatter plot (Fig. 3a). Patients with a high level of miR-96 showed shorter OS and disease-free survival (DFS) than those with low miR-96 in the Kaplan–Meier plot (Fig. 3b). A low level of *PTEN* was also associated with shorter OS and DFS (Fig. 3c). Notably, patients with high miR-96 and low *PTEN* presented shorter OS and DFS (Fig. 3d). In all three cases, the fact that *P* values of OS were lower than those of DFS indicated the clinical significance of miR-96 and PTEN expression.

**Figure 4:**
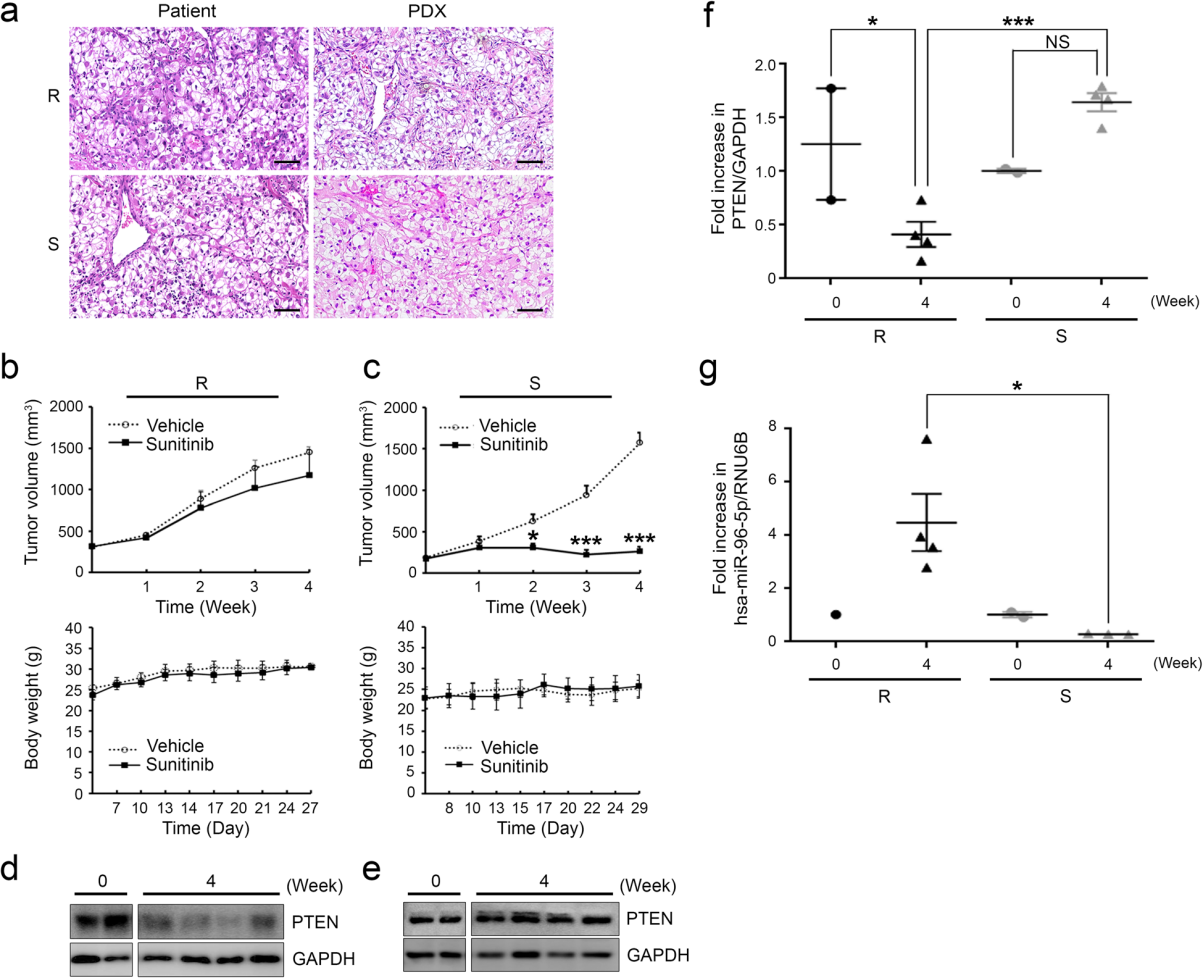
Modulation of sunitinib sensitivity by the miR-96-5p/PTEN axis in CCRCC patient-derived xenografts (PDXs). (**a**) H&E staining images of tumors from original sunitinib-resistant and sunitinib-sensitive patients and their corresponding PDXs (scale bar = 50 μm). (**b**, **c**) PDX models were divided into two treatment groups: vehicle and 40 mg/kg sunitinib. Body weight and tumor volumes were assessed for changes over time (Vehicle n = 4; sunitinib n = 4, mean ± s.e.m., *P < 0.05, ***P < 0.001, one-way ANOVA). (**d**, **e**) Comparison of the expression of PTEN between sunitinib-resistant (d) and sunitinib-sensitive PDX (e) at 0 and 28 days; GAPDH, internal control. (**f, g**) PTEN (f) and miR-96-5p (g) expression from tissue of sunitinib-resistant PDX, compared with the sunitinib-sensitive counterparts (mean ± s.e.m., *P < 0.05, ***P < 0.001, one-way ANOVA, n = 3).

Next, we investigated the in vivo effects of miR-96-5p on PTEN in tumors of two PDXs representing different responses to sunitinib. Tumor tissues were implanted subcutaneously into SCID mice and grown tumors were transferred to athymic nude mice for an experiment on the inhibitory effects of sunitinib on tumor growth. Hematoxylin and eosin staining of the tumors showed that histopathological features of the corresponding CCRCC patients were retained in the tumors of PDX (Fig. 4a). When we evaluated the anti-cancer effect of sunitinib in these two CCRCC PDX models, oral administration of 40 mg/kg sunitinib for 4 weeks represented hugely different responses (Fig. 4b, c). The tumor volume was notably reduced only in sensitive PDX (Fig. 4c), but not in the resistant model, in response to sunitinib treatment (Fig. 4b). The body weights of mouse were not changed by sunitinib treatment in both PDXs (Fig. 4b, c). We hypothesized that the levels of PTEN and miR-96-5p differed in these PDXs and then explored their expression. The expression of PTEN protein was downregulated in tumors of resistant PDX 4 weeks after the administration of sunitinib compared with that before treatment (Fig. 4d, f), but this was not the case in sensitive PDX (Fig. 4e, f). Comparably, the levels of miR-96-5p were highly elevated only in tumors from resistant PDX at 4 weeks after sunitinib treatment (Fig. 4g). These results demonstrated the possibility that the dysregulation of PTEN by high levels of miR-96-5p induces sunitinib resistance in CCRCC.

### Direct downregulation of PTEN by miR-96-5p decreases sensitivity to sunitinib in CCRCC cell lines

**Figure 5:**
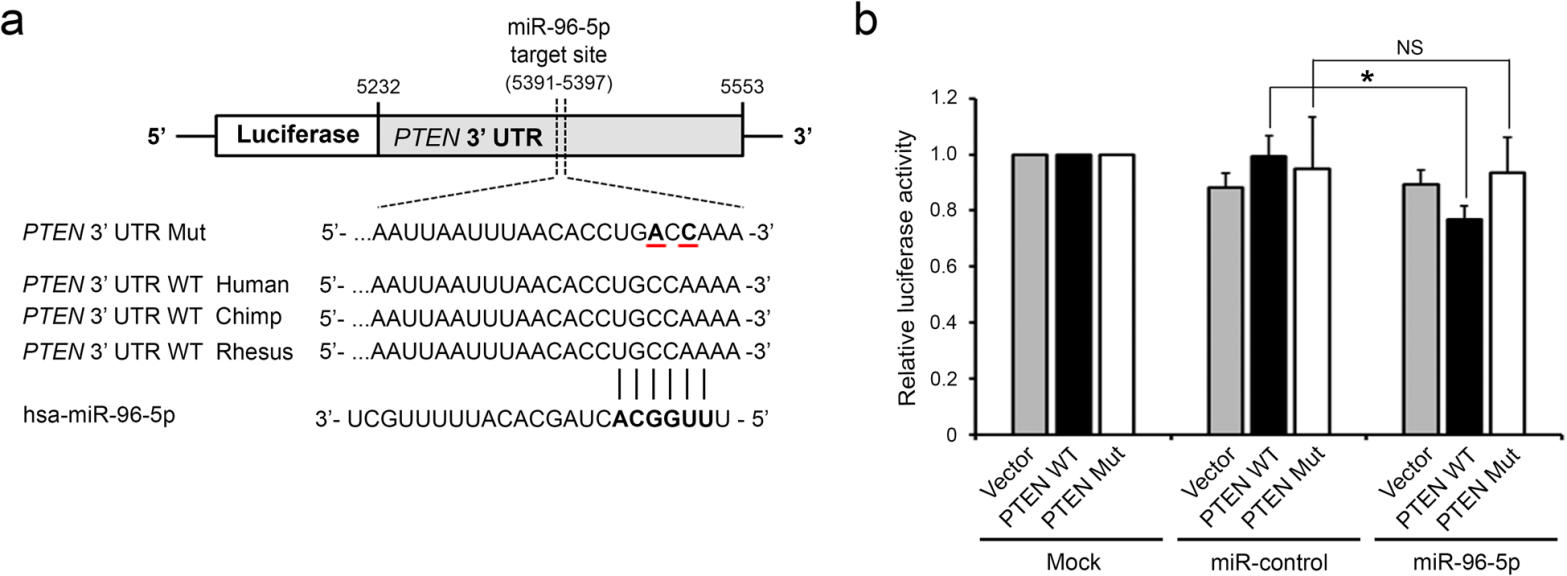
miR-96-5p directly represses PTEN expression. (**a**) Schematic representation of the predicted miR-96-5p target site sequence within the 3′ UTR of *PTEN*. Six nucleotides within the target site are complementary to the seed sequence of miR-96-5p. (**b**) Luciferase reporter assay. 293 T cells were co-transfected with luciferase reporters carrying either the wild-type *PTEN* 3′ UTR (PTEN WT) or the mutagenized *PTEN* 3′ UTR (PTEN Mut), as well as 50 nM negative control mimic (miR-Control) or miR-96-5p mimic. Data are presented as mean ± s.e.m. (n = 3). Significant results are presented as *P < 0.05.

Next, we determined whether miR-96-5p could directly repress *PTEN* mRNA, leading to sunitinib resistance. Two independent groups have recently reported that PTEN is directly regulated by miR-96-5p in head and neck squamous cell carcinoma (HNSCC)^15^ and cervical cancer^14^. We found an additional regulatory site in the 3′ UTR of *PTEN* with a possible miR-96-5p binding site. Interestingly, the seed sequences of the miR-96-5p binding site within *PTEN* 3′ UTR at positions 5391–5397 showed conservation across most vertebrates including primates (Fig. 5a). The partial *PTEN* 3′ UTR with an miR-96-5p site was cloned downstream of the *Renilla* luciferase reporter (Fig. 5a). The overexpression of miR-96-5p inhibited reporter expression by 23% (Fig. 5b, black bars). Notably, this repression was sequence-specific; a mutant reporter construct with mutations of two nucleic acids in the miR-96-5p binding site failed to show any suppression (Fig. 5b, white bars).

**Figure 6:**
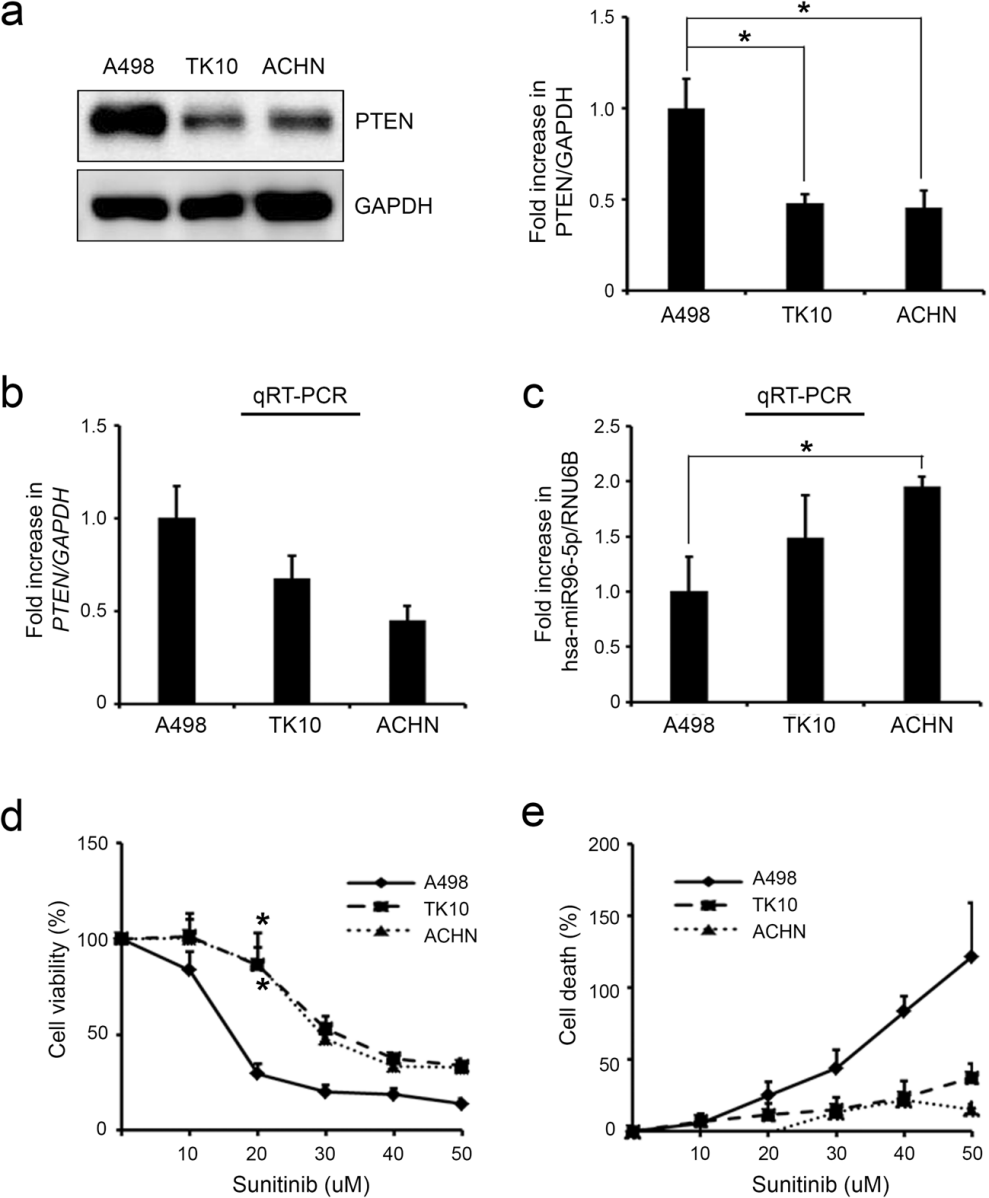
Downregulation of PTEN and upregulation of miR-96-5p are associated with sunitinib resistance in CCRCC cell lines. (**a**) Western blot (left) and quantification (right) of PTEN proteins in A498, TK10, and ACHN cells. GAPDH was used as a loading control. (**b**, **c**) The levels of PTEN mRNA (**b**) and miR-96-5p (**c**) were analyzed by qRT-PCR in A498, TK10, and ACHN cells. All data were normalized with GAPDH for mRNA or RNU6B for miRNA. (**d**, **e**) Dose-dependent effect of sunitinib on the viability (**d**) and death (**e**) of A498, TK10, and ACHN cells. Data are presented as mean ± s.e.m. (n = 3). Significant results are presented as *P < 0.05.

**Figure 7:**
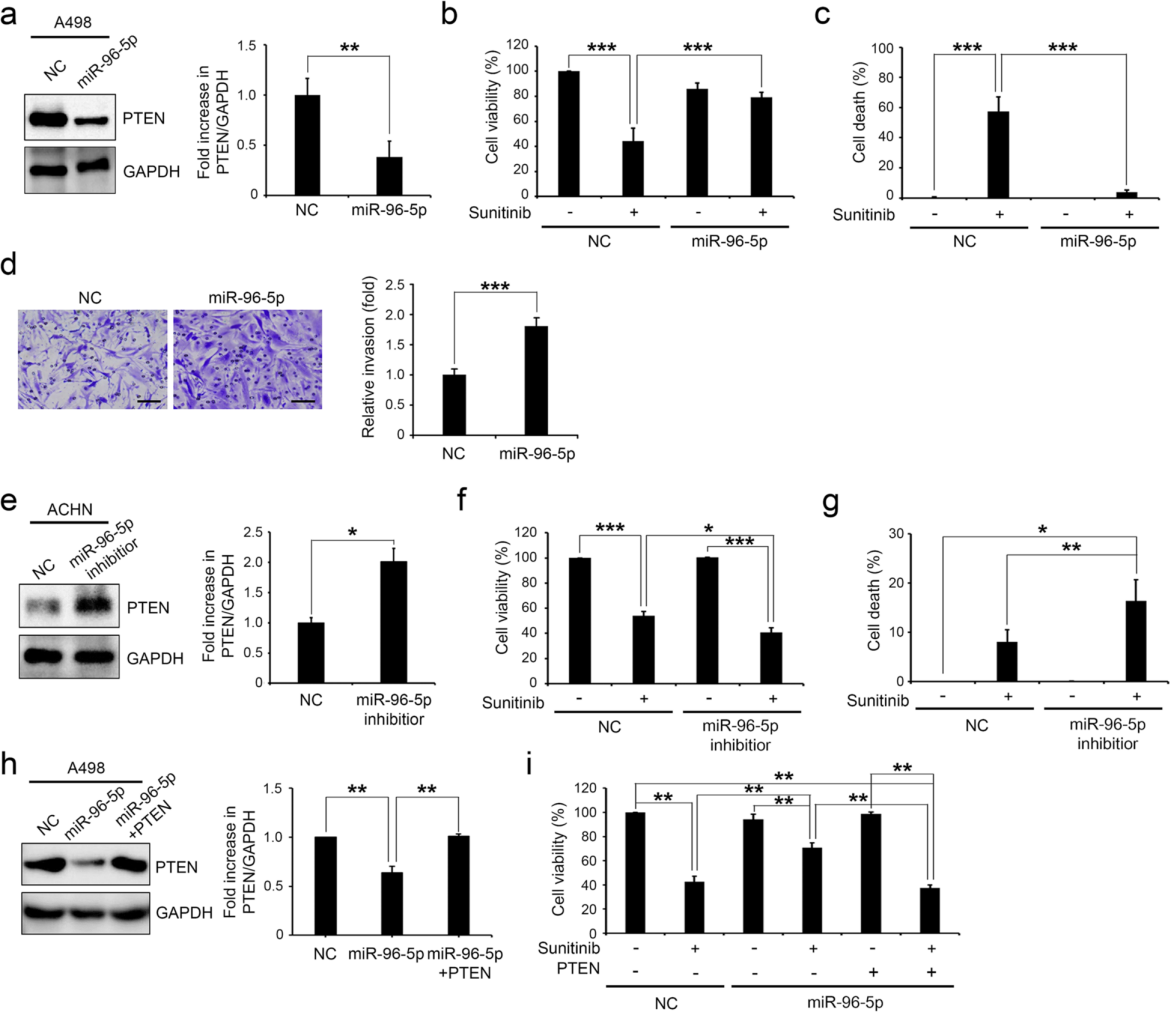
miR-96-5p-mediated downregulation of PTEN regulates sensitivity to sunitinib in CCRCC cell lines. (**a**) The expression level of PTEN was downregulated after overexpression of miR-96-5p mimic (miR-96-5p) in the A498 cell line. (**b**, **c**) PTEN downregulation affects the viability (**b**) and death (**c**) of A498 cell lines upon treatment with 20 μM sunitinib. (**d**) Representative images of Matrigel invasion assay of NC and miR-96-5p transfected A498 cells under 10 uM sunitinib treatment (scale bars, 100 μm). The number of invaded cells was quantified and statistically analyzed (***P < 0.001). Error bars indicate the standard error of mean of four experiments. (**e**) The expression level of PTEN was upregulated after knockdown of miR-96-5p in the ACHN cell line. (**f**, **g**) The effects of PTEN upregulation on the viability (**f**) and death (**g**) upon 20 μM sunitinib treatment in ACHN. (**h**) The expression level of PTEN was verified after overexpression of PTEN in the miR-96-5p-expressing A498 cell line. (**i**) Upregulation of PTEN significantly sensitized cells to sunitinib treatment in miR-96-5p-expressing A498. Data are presented as mean ± s.e.m. (n = 3). Significant results are presented as *P < 0.05, **P < 0.01, ***P < 0.001. NC: negative control.

To confirm the induction of resistance to sunitinib via repressing PTEN expression by miR-96-5p in CCRCC cell lines, we first analyzed the levels of PTEN and miR-96-5p in A498, TK10, and ACHN kidney cancer cell lines. The results of Western blot and qRT-PCR showed that the level of PTEN expression was higher in A498 cells than in TK10 and ACHN cells (Fig. 6a, b). In contrast, the level of miR-96-5p was inversely correlated with PTEN expression in A498, TK10, and ACHN cells (Fig. 6c). These cell lines were exposed to 20 μM sunitinib for 24 h, after which we measured the cell viability and death. Interestingly, A498 cells, which showed high PTEN and low miR-96-5p levels, were highly sensitive to sunitinib compared with TK10 and ACHN (Fig. 6d, e). Next, we manipulated the expression of miR-96-5p by infecting RCC cell lines with lentiviruses expressing either miR-96-5p mimic or miR-96-5p inhibitor and then assessed the PTEN levels and sunitinib sensitivity by evaluating various cellular phenotypes. Infection of miR-96-5p-expressing lentivirus decreased PTEN proteins significantly in A498 cells (Fig. 7a) and made the cells resistant to sunitinib by increasing cell viability and decreasing cell death (Fig. 7b, c). In addition, the overexpression of miR-96-5p significantly increased the invasion ability of A498 cells in the presence of sunitinib (Fig. 7d). In contrast, infection of ACHN cells with miR-96–5-p inhibitor increased PTEN expression (Fig. 7e) and enhanced sensitivity to sunitinib by decreasing cell viability and increasing cell death (Fig. 7f, g). In addition, after reestablishment of the levels of PTEN in miR-96-5p-expressing A498 cells by infection with PTEN-expressing lentivirus (Fig. 7h), the sensitivity to sunitinib was restored (Fig. 7i). Taking these findings together, the downregulation of PTEN directly by miR-96-5p in part contributed to the resistance to sunitinib in CCRCC cell lines.

## Discussion

Although the multi-kinase inhibitor sunitinib has benefits as a therapeutic for CCRCC by extending the survival period, the development of resistance is a serious problem in patients with CCRCC. There is thus a need to understand the mechanisms underlying drug resistance and find a biomarker predictive of it. Emerging evidence has suggested that miRNAs regulate the progression of cancer, including CCRCC. In the present study, we identified a novel regulatory network of miR-96-5p and PTEN responsible for tumor progression and resistance to sunitinib in CCRCC. Using miRNA profiling, we found that miR-96-5p was significantly increased in sunitinib-resistant tumors compared with the level in sensitive tumors from CCRCC patients and confirmed its expression in tumors from a CCRCC PDX model. The expression level of miR-96-5p was significantly upregulated only in the resistant PDX model, representing different responses to sunitinib depending on the miR-96-5p expression. We found a novel miR-96-5p binding site in the 3′ UTR of *PTEN* and demonstrated its direct repression by luciferase reporter assays. Supporting this, upon the overexpression of mimics or inhibitors of miR-96-5p, there was a negative correlation of PTEN with miR-96-5p expression in various CCRCC cell lines. Consistent with this, the level of PTEN was inversely associated with miR-96 in 514 patients of the KIRC cohort in TCGA data and a resistant PDX model. To further examine the clinical implications of high miR-96 and low *PTEN* levels, we analyzed the survival in the KIRC cohort, although the patients were treated with different kinds of anti-cancer drugs including VEGFR inhibitors (sunitinib, sorafenib, or pazopanib) and rapalogues (sirolimus or temsirolimus). Intriguingly, OS as well as DFS was longer in the patients with low miR-96 and high *PTEN* levels compared with those with high miR-96 and low *PTEN*. In the patient samples used for miRNA profiling and PDX models, high miR-96-5p and low PTEN were closely associated with resistance to sunitinib. A negative correlation of miR-96-5p and PTEN expression was also observed in various CCRCC cell lines. Taken together, these results raise the possibility that high levels of miR-96-5p repress PTEN, which is in part responsible for sunitinib resistance and poor prognosis in CCRCC.

The overexpression of oncogenic miR-96-5p has been reported in various types of cancer, including prostate, ovarian, endometrial, cervical, breast, and head and neck cancer^10–15^. In these studies, miR-96-5p was shown to target tumor suppressor genes such as forkhead box O1 (FOXO1) in endometrial, prostate, and bladder cancer^11,13,16^ and protein tyrosine phosphatase non-receptor type 9 (PTPN9) in breast cancer^10^, promoting cell proliferation, migration, and clonogenicity. In CCRCC, there are contradictory reports about the role of miR-96-5p. Yuan et al. demonstrated that the miR-183/182/96 cluster consists of carcinogenic miRNAs and is a useful predictor of prognosis in CCRCC^17^. In contrast, miR-96-5p was also suggested as a tumor repressor^18,19^. Consistent with the results of Yuan et al., our results support the oncogenic role of miR-96-5p in CCRCC. In particular, we suggest that the downregulation of PTEN by miR-96-5p is a novel mechanism involved in drug resistance. Taking these findings together, we propose miR-96-5p as a possible predictive factor for sunitinib resistance in CCRCC.

PTEN, a tumor suppressor, acts as a negative regulator of the phosphatidylinositol 3-kinase/AKT/mTOR pathway, playing a critical role in tumorigenicity^20^. Therefore, the loss of PTEN increases the aggressiveness of tumors and resistance to many tyrosine kinase inhibitors including sunitinib. The positive correlation of PTEN with sunitinib sensitivity has been reported in advanced RCC. It was demonstrated that the expression of PTEN sensitized cells to sunitinib^21^ and its knockout using CRISPR-Cas9 led to resistance to sunitinib in CCRCC cell lines^22^. It was also shown that PTEN positivity had value in predicting a good response to sunitinib in immunohistochemical analysis of tumor tissues from RCC patients^22,23^. Moreover, it was reported that long-term treatment with sunitinib silenced PTEN expression via methylation of the promoter in gastrointestinal stromal tumor (GIST)^24^. Consistent with these previous reports, we demonstrated that high levels of PTEN were related to sensitivity to sunitinib in tumors from CCRCC patients, PDX models, and CCRCC cell lines. We acquired tumors used in our experiments in two different ways. One was during surgery from patients who had never been exposed to sunitinib, with these tumors being used for an miRNA microarray experiment. The other was from tissues of PDXs treated with sunitinib for 4 weeks. The former patients were subsequently treated with sunitinib when recurrence occurred. Interestingly, upregulation of miR-96-5p and downregulation of PTEN were observed in sunitinib-resistant tissues from both methods, indicating that the repression of PTEN expression by miR-96-5p could be the cause of de novo and acquired sunitinib resistance.

The oncogenic activities of miR-96-5p by repressing PTEN have recently been reported in other cancers^14,15^. For example, Vahabi et al. reported that the level of miR-96-5p was high in HNSCC patients, especially in p53 mutated HNSCC, which was associated with shorter recurrence-free survival^15^. Using head and neck cancer cell lines, they showed that the overexpression of miR-96-5p downregulated PTEN, which led to chemo-radioresistance by promoting cell migration without changes in cell proliferation. In addition, Shao et al. demonstrated that PTEN-targeting miR-96-5p is suppressed by a long noncoding RNA, STXBP5-AS1. The STXBP5-AS1/miR-96-5p/PTEN axis was shown to be closely related to cancer cell proliferation and migration in cervical cancer cell lines^14^. High miR-96-5p and low STXBP-AS1 were correlated with poor OS in cervical cancer. Consistent with this, here we showed that miR-96-5p directly represses PTEN, and accelerates cell proliferation, migration, and resistance to sunitinib, leading to poor prognosis in CCRCC. However, there were discrete cognate sites of miR-96-5p in the 3′ UTR of *PTEN* in all three cases. Vahabi et al. demonstrated that positions 3702–3720 of the *PTEN* 3′ UTR are an miR-96-5p binding site, while Shao et al. did the same for positions 6385–6401. We found an additional seed sequence motif (5391–5397) for direct miR-96-5p binding. The differences between the distinct binding sites might be due to the various target prediction programs used in each study. Interestingly, the miR-96-5p binding sites that we demonstrated are broadly conserved among vertebrates including apes, suggesting an evolutionarily conserved regulatory effect by miR-96-5p. The existence of multiple target sites of miR-96-5p on PTEN, one of the most important tumor suppressors, implies the complexity of how tumors acquire resistance to chemo- and radiotherapies.

Taken together, our findings suggest that PTEN expression is negatively associated with the levels of miR-96-5p and that miR-96-5p and PTEN are highly related to sunitinib treatment response and prognosis in CCRCC. Considering the multiple and complex regulation of miRNAs and their target mRNAs, the regulatory axis consisting of miR-96-5p and PTEN in CCRCC is in part responsible for sunitinib resistance. Because the drug resistance is acquired by diverse mechanisms, additional regulatory circuits related to it remain to be identified. Although, more intensive and extended study is needed in CCRCC patients treated with sunitinib, our findings raised the possibility to use miR-96-5p and PTEN as biomarkers for predicting drug sensitivity. This strategy can facilitate the implementation of the precision medicine by determining an optimal therapy for each individual CCRCC patient.

## Methods

### Tumor samples and miRNA microarray

The frozen human CCRCC tumors and adjacent normal kidney tissues used in this study were provided by Asan Bio-Resource Center (AMC, Seoul, Republic of Korea) and Korea Biobank Network after approval by the Institutional Review Board of AMC (approval No. 2012–0872). miRNA microarray and data analyses were performed as previously described^25,26^ using Agilent Human Microarray 8 × 60 K Release 19.0 (Agilent Technologies, Santa Clara, CA). Briefly, total RNA was extracted using Trizol (Invitrogen, Carlsbad, CA), in accordance with the manufacturer’s instructions and treated with DNase I (Fermentas, Waltham, MA). RNA quality was evaluated using the Agilent 2100 Bioanalyzer. One hundred nanograms per sample was hybridized to the microarray. We compared the expression profiles of 11 samples (2 normal sensitive, 3 normal resistant, 3 tumor sensitive, and 3 tumor-resistant samples). miRNA labeling, hybridization, and washing were performed in accordance with the manufacturer’s instructions. Sixty-eight differentially expressed miRNAs were identified by ANOVA *t*-test (*P* < 0.05) and selected hierarchical clustering.

### Target prediction and functional analysis

The target genes of miRNAs were predicted by TargetScan 7.2. Each predicted target includes a poorly conserved site. Among the total targets of miRNAs, a list of genes commonly targeted by more than six differentially expressed miRNAs in resistant samples was established. These filtered target genes were then analyzed by IPA software (Qiagen, Hilden, Germany) and run for core analysis including canonical pathways, networks, and disease and biofunctions were generated through the use of IPA (QIAGEN Inc., https://www.qiagenbioinformatics.com/products/ingenuity-pathway-analysis)^27^.

### RT-PCR and real-time qPCR

Total RNA was extracted from cells and tissues using miRNeasy Mini kit (Qiagen), in accordance with the manufacturer’s instructions. The first-strand cDNA was synthesized from 1 to 2 μg of total RNA using SuperScript III Reverse Transcriptase (Invitrogen). After synthesis, real-time PCR was performed using the RT^2^ SYBR Green qPCR Mastermix (Qiagen) and Roche LightCycler 480 (96-well Block), in accordance with the manufacturer’s instructions. Primers used in RT-qPCR were PTEN-F 5′-CGACGGGAAGACAAGTTCAT-3′, PTEN-R 5′-AGGTTTCCTCTGGTCCTGGT-3′, GAPDH-F 5′-CAAGATCATCAGCAATGCCT-3′, and GAPDH-R 5′-ATGGACTGTGGTCATGAGT-3′. The amplification data (fold changes in Ct values of all of the genes) were analyzed using the ΔΔCt method.

### Dual-luciferase reporter assay

The miR-96-5p binding site in the 3′ UTR of *PTEN* was predicted by TargetScan7.2, the miRNA target prediction database. To construct the luciferase reporter, the *PTEN* 3′ UTR with an miR-96-5p binding site and XhoI/NotI recognition sites was synthesized and first cloned in the pUCosmo_Amp vector (Cosmogenetech, Seoul, Republic of Korea). The fragment was then cut and transferred to the psiCHECK2 vector for the assay. The plasmid containing *PTEN* 3′ UTR, miRNA control, and miR-96-5p mimic (Dharmacon, Lafayette, CO) was transfected into 293 T cells using Fugene (Promega, Madison, WI) and Dharmafect reagent (Dharmacon). After incubation of HEK293T cells at 37 °C for 24 h, luciferase activity was measured using a Dual-Luciferase Reporter Assay system (Promega). *Renilla* luciferase activity was normalized to the firefly luciferase activity of each sample.

### Invasion assay

Cell migration and invasion were assessed using invasion assay. Briefly, cell culture inserts (24-well format, 8.0 μm pore size) were pre-coated with Matrigel (1:5 dilution; Corning). Transfected cells were then seeded at 10^5^ cells/insert in a serum free medium. The complete medium supplemented with 10% FBS was added to the lower chamber. All medium was applied with 10 μM sunitinib. After 48 h incubation, cell culture inserts were fixed with 100% methanol and stained with 0.3% crystal violet solution. The cells in the upper chamber were then removed with cotton swab and the inserts were mounted on glass slides. Microscopic images were acquired using an Eclipse Ts2-FL inverted microscope (Nikon) and the numbers of cells were counted using ImageJ software.

### PDX for CCRCC

We followed the protocol approved by the Institutional Animal Care and Use Committee of Asan Medical Center. Tumor tissue of 1 to 3 mm^3^ from CCRCC was implanted subcutaneously into the flank of 5-week-old male athymic nude mice (Central Lab. Animal Inc., Seoul, Republic of Korea). When the tumor volume reached 200 to 300 mm^3^, 40 mg/kg sunitinib in 0.1 M citrate buffer was orally administered for 5 days per week for 4 weeks. Tumor volume was measured using calipers and calculated using the following formula: length × width^2^ × 0.5 (length = longest diameter across the tumor, width = corresponding perpendicular diameter).

### Cell culture and lentiviral infection

A498, TK10, and ACHN cell lines (National Cancer Institute, Bethesda, MD) were cultured in RPMI-1640 medium (Invitrogen) containing 10% fetal bovine serum and 100 μg/ml penicillin–streptomycin at 37 °C in an atmosphere of 5% CO_2_. The medium was changed every 2–3 days and cells at passages 4–8 were used. Cells were treated with sunitinib obtained from LC Laboratories (LC Laboratories, Woburn, MA) in growth medium.

To manipulate the expression of miR-96-5p and PTEN in cells, we purchased lentiviral miRNA-96 (pLV-hsa-mir-96 plasmid, mir-p069), miR-96-5p inhibitor (pLV-hsa-miR-96-5p locker plasmid, mir-hp0066), mir-control lentivirus (mir-LV000) from Biosettia (San Diego, CA), and PTEN lentivirus (#51304) from Addgene (Watertown, MA). A498 or ACHN cells were infected with 3 ml of 1.65 × 10^4^ TU/ml lentiviruses overnight in the presence of 4 μg/ml polybrene. Medium was replaced 16 h after infection. Permanent cell lines expressing miR-96-5p and miR-96-5p inhibitor were selected by the addition of 1 μg/ml puromycin or 400 ng/ml blasticidin, respectively. PTEN was transiently expressed in miR-96-5p-expressing A498 cells.

### Western blotting

CCRCC cells were lysed in buffer containing 50 mM Tris–HCl (pH 7.5), 150 mM NaCl, 0.1% (w/v) SDS, 0.1% (w/v) SDC, 1% (v/v) Nonidet P-40, 5 mM NaF, 1 mM Na_3_VO_4_, 2.5 mM Na_2_HPO_4_, and protease inhibitor cocktail (Roche Diagnostics, Basel, Switzerland) and tissues were lysed in Pro-prep buffer (Intron, Daejeon, Korea). Equal amounts of proteins were separated by SDS–polyacrylamide gel electrophoresis and transferred to polyvinylidene difluoride membranes (Millipore, Bedford, MA). Membranes were probed with antibodies to PTEN (Cell Signaling, 91885) and GAPDH (sc-20357, Santa Cruz Biotechnology, Inc.), followed by incubation with the appropriate secondary antibody conjugated to horseradish peroxidase (ThermoFisher Scientific, Waltham, MA). Immunoreactivity was visualized using Immunobilon Western Chemiluminescent HRP Substrate (Millipore) and the Amersham Imager 600.

### Cell viability and death assay

Metabolically active cells were quantified by measuring the ATP level using CellTiter-Glo reagent (Promega), in accordance with the manufacturer’s instructions. Cell viability was determined by measuring the luminescence signal with an EnVision Multilabel Reader (Perkin-Elmer, Waltham, MA). Cell death was measured by analyzing lactate dehydrogenase (LDH) released into culture medium by damaged cells. The mean background value in control sister cultures that only underwent a sham wash (0% cell death) was subtracted from the LDH value in each test condition.

### Gene expression data analysis

Gene expression data are publicly available from the National Center for Biotechnology Information Gene Expression Omnibus database (http://www.ncbi.nlm.nih.gov/geo) and TCGA data portal site (https://tcga-data.nci.nih.gov/tcga/). All data were processed using Biometric Research Branch array tools. Patients were discriminated into indicated groups depending on the gene expression values according to the Cutoff finder under R program and then applied for Kaplan–Meier plot.

### Statistical analysis

Data were obtained from at least three independent experiments and are presented as mean ± s.e.m. Statistical evaluation of the results was performed using one-way ANOVA. Data were considered significant at *P < 0.05.

## Supplementary Information

Supplementary Information 1.

Supplementary Information 2.

## Data Availability

All microarray data are available at the Gene Expression Omnibus (GEO) database with accession number GSE189331.
