# Supplementary material for: miR-96-5p targets PTEN to mediate sunitinib resistance in clear cell renal cell carcinoma
Source: Sci Rep. 2022 Mar 3;12:3537. doi: 10.1038/s41598-022-07468-x (PMC8894382; doi:10.1038/s41598-022-07468-x)
Supplement: Supplementary file 1 — Supplementary Information 1. [file 41598_2022_7468_MOESM1_ESM.docx]

**Supplementary Information**

**miR-96-5p targets PTEN to mediate sunitinib- resistance in clear cell renal cell carcinoma**

Sang Eun Park^1^#, Wonju Kim^2,3^#, Ji-Ye Hong^1^, Dayeon Kang^3^, Seulki Park^1,4^, Jungyo Suh^5^, Dalsan You^5^, Yun-Yong Park^6^, Nayoung Suh^2,3^* Jung Jin Hwang^1^*, Choung-Soo Kim^5^*

*^1^Asan Institute for Life Sciences, Asan Medical Center, Seoul, 05505, Republic of Korea*

*^2^Department of Pharmaceutical Engineering, College of Medical Sciences, Soon Chun Hyang University, Asan, 31538, Republic of Korea*

*^3^Department of Medical Sciences, General Graduate School, Soon Chun Hyang University, Asan, 31538, Republic of Korea*

*^4^Disease Target Structure Research Center, Korea Research Institute of Bioscience and Biotechnology (KRIBB), Daejeon, 34141, Republic of Korea*

*^5^Department of Urology, University of Ulsan College of Medicine, Asan Medical Center, Seoul, 05505, Republic of Korea*

*^6^ Department of Life Science, Chung-Ang University, Seoul, 06911, Republic of Korea*

**Fig. S1.** A heat map of 345 reliable genes of tumor and adjacent noncancerous tissues from sunitinib resistant and sensitive CCRCC patients was constructed using Orange (<https://orangedatamining.com/>).^1^


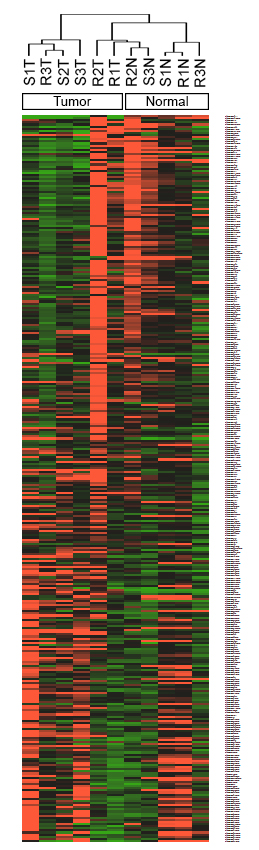


**Fig. 2b. Original blots**

**
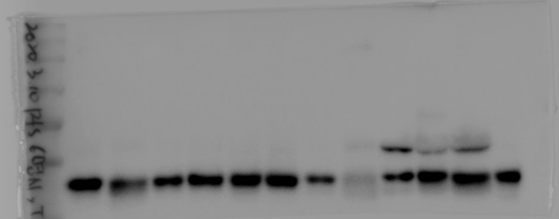
**

PTEN

GAPDH

R1 R2 R3 S1 S2 S3

Tumor

**
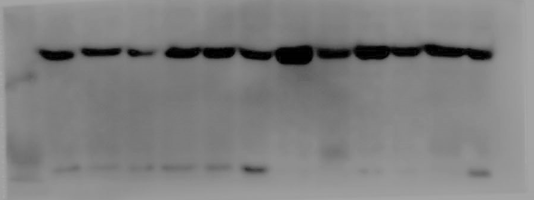
**

Tumor

**
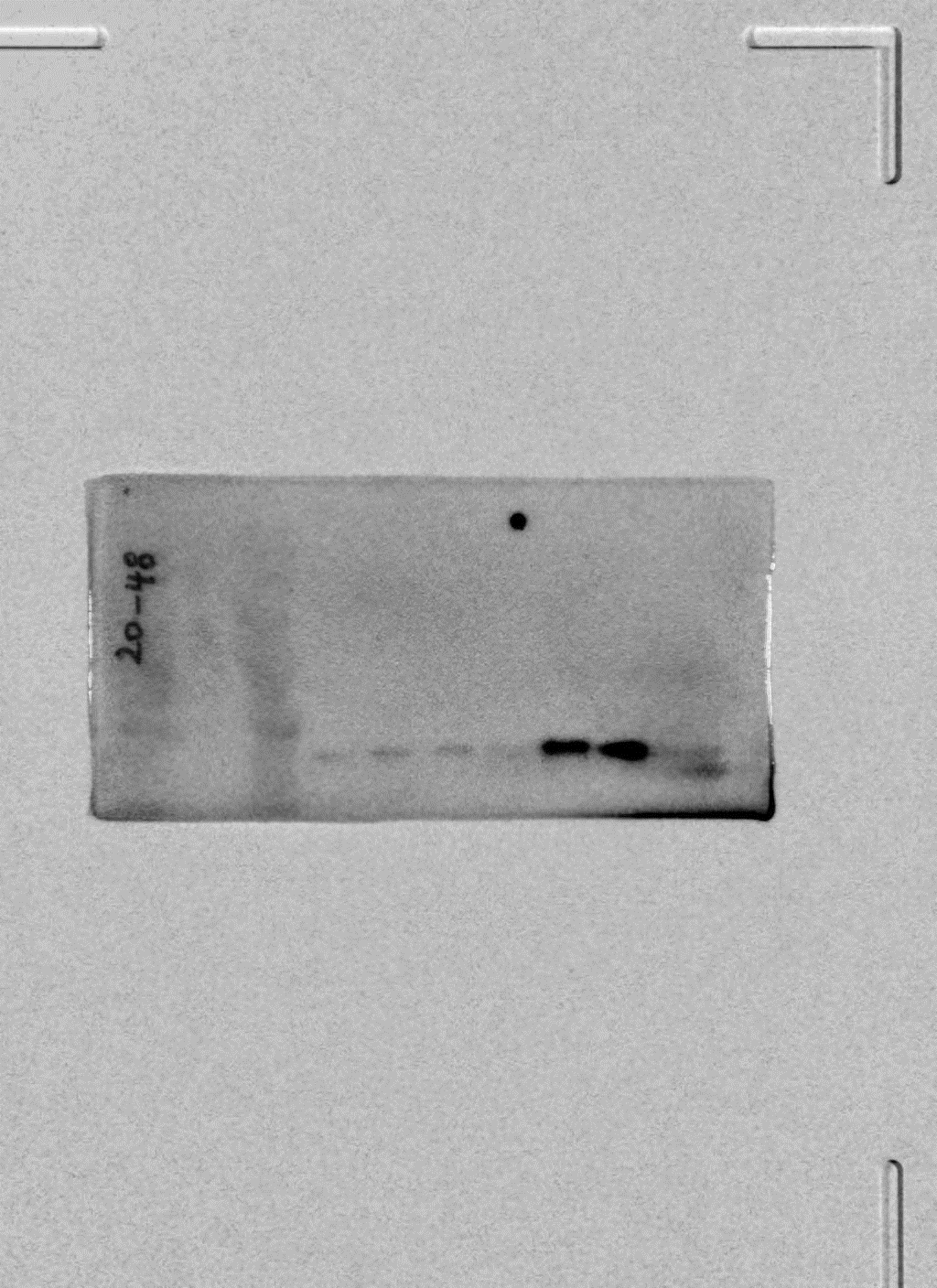

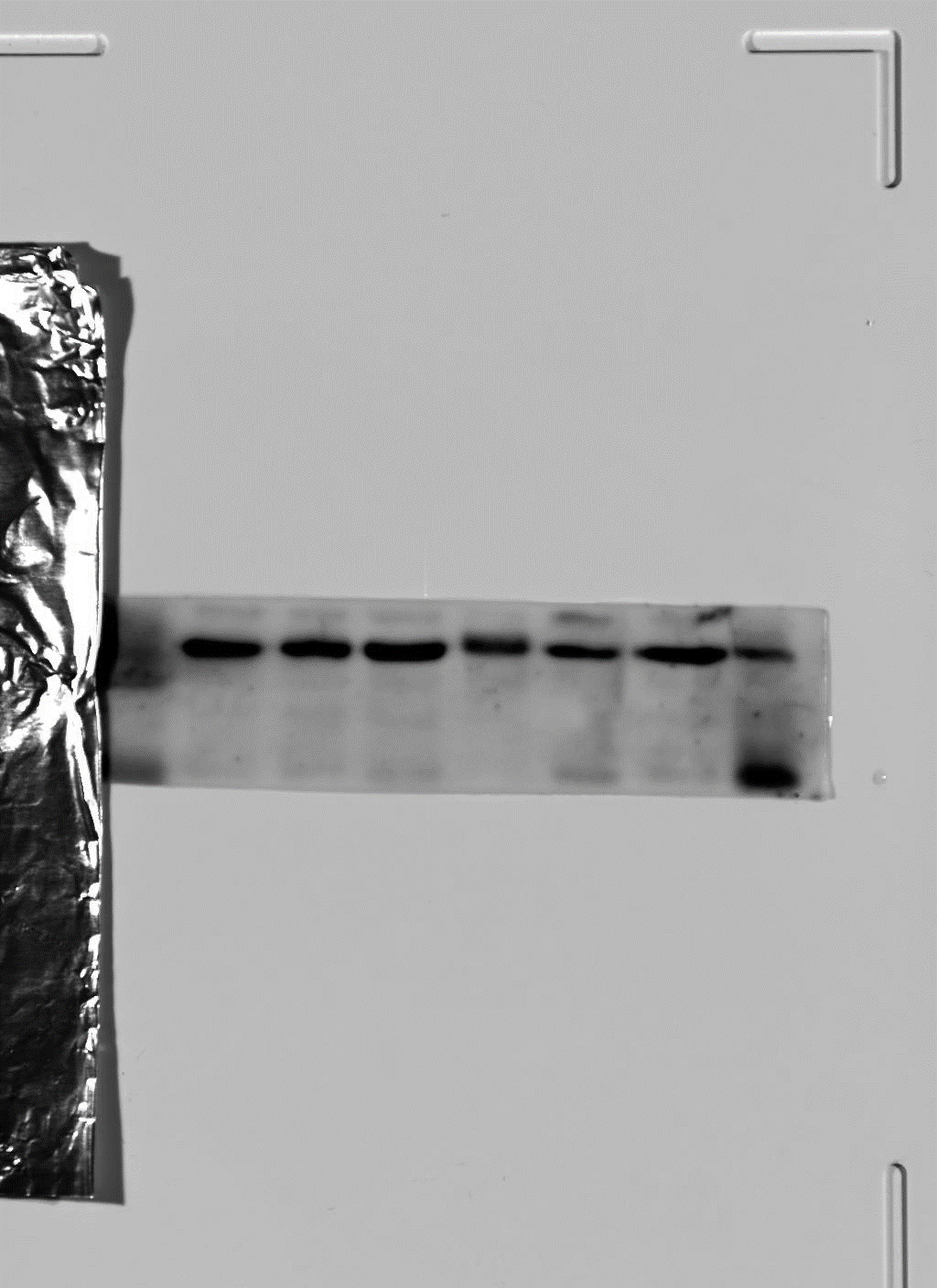
**

R1 R2 R3 S1 S2 S3

GAPDH

PTEN

**Fig. 4d, 4e. Original blots**

**
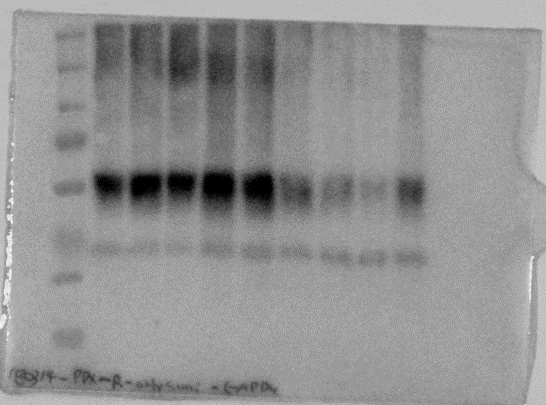

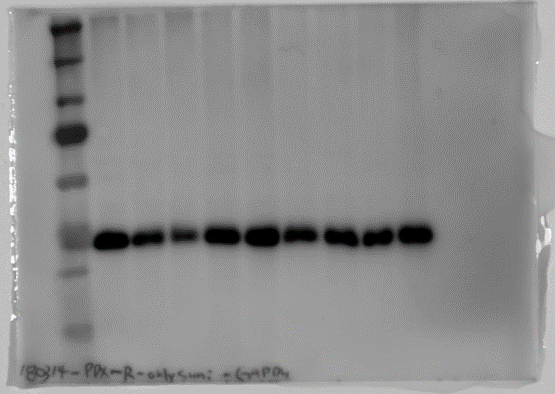

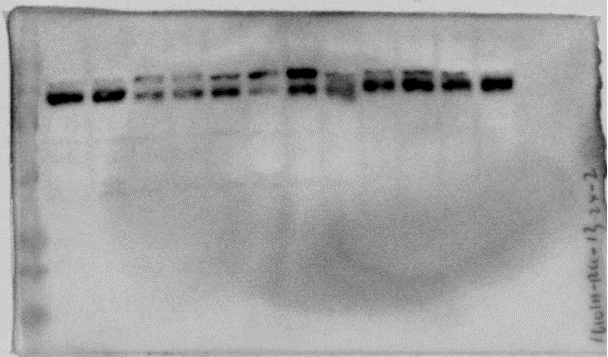

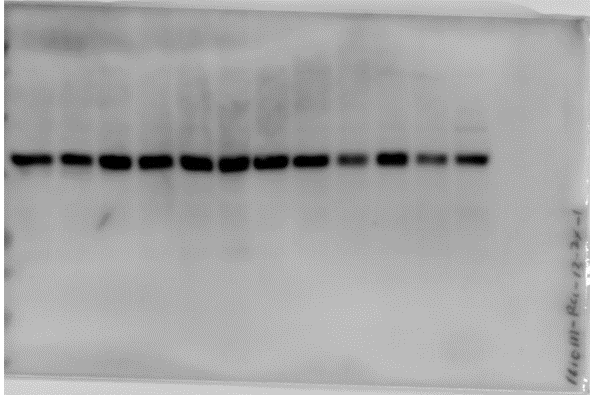
**

PTEN

(resistance)

GAPDH

(resistance)

PTEN

(sensitive)

GAPDH

(sensitive)

0 week

4 week

0 week

4 week

**Fig. 6a. Original blots**

**
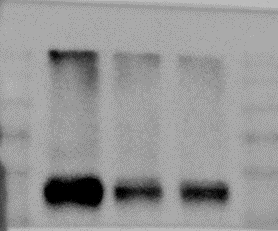

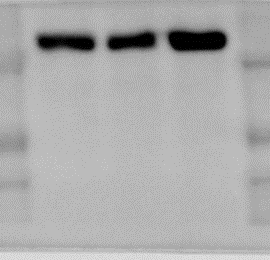
**

PTEN

GAPDH

A498 TK10 ACHN

**
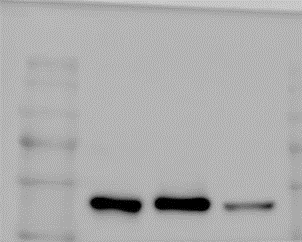
Fig. 7a, 7e, 7h. Original blots**

**
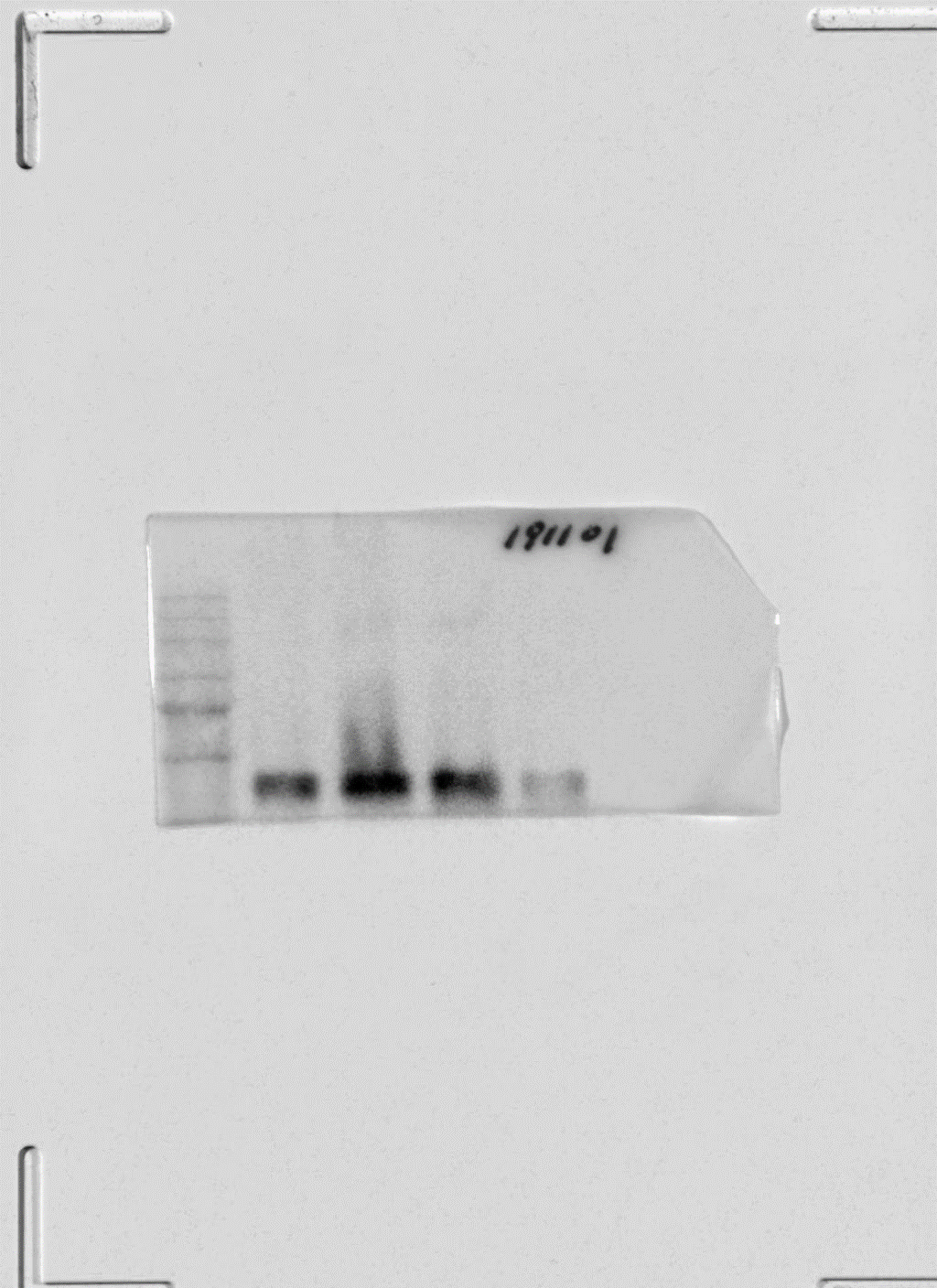

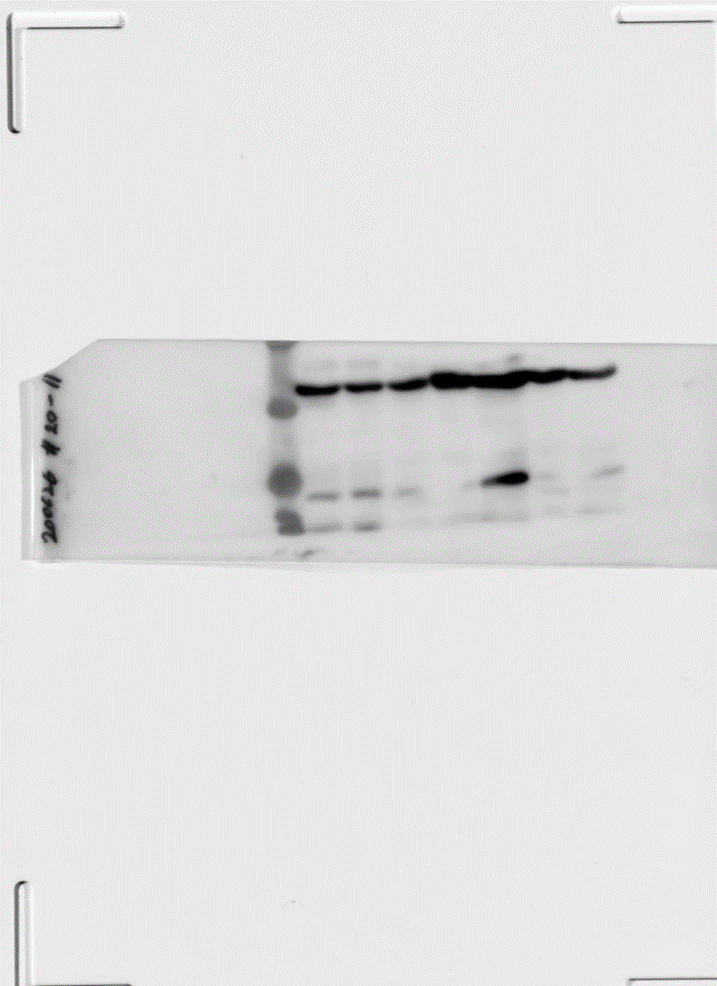

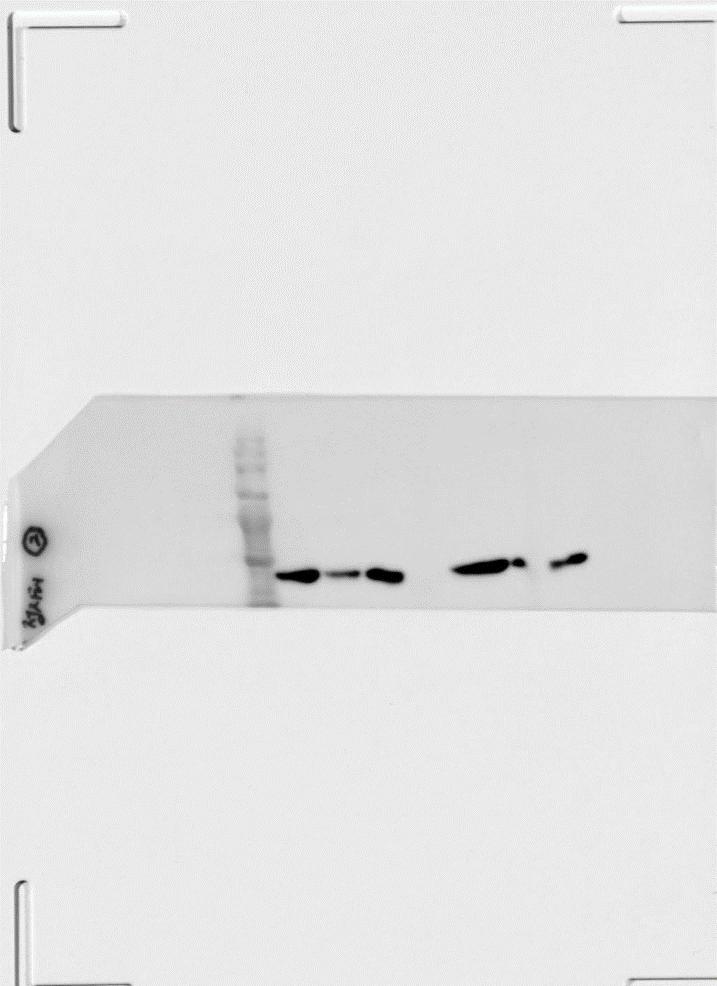

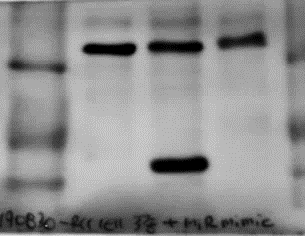
**

miR-96-5p

inhibitor

NC

miR-96-5p

+PTEN

miR-96-5p

NC

miR-96-5p

PTEN

GAPDH

PTEN

GAPDH

PTEN

GAPDH

NC

**
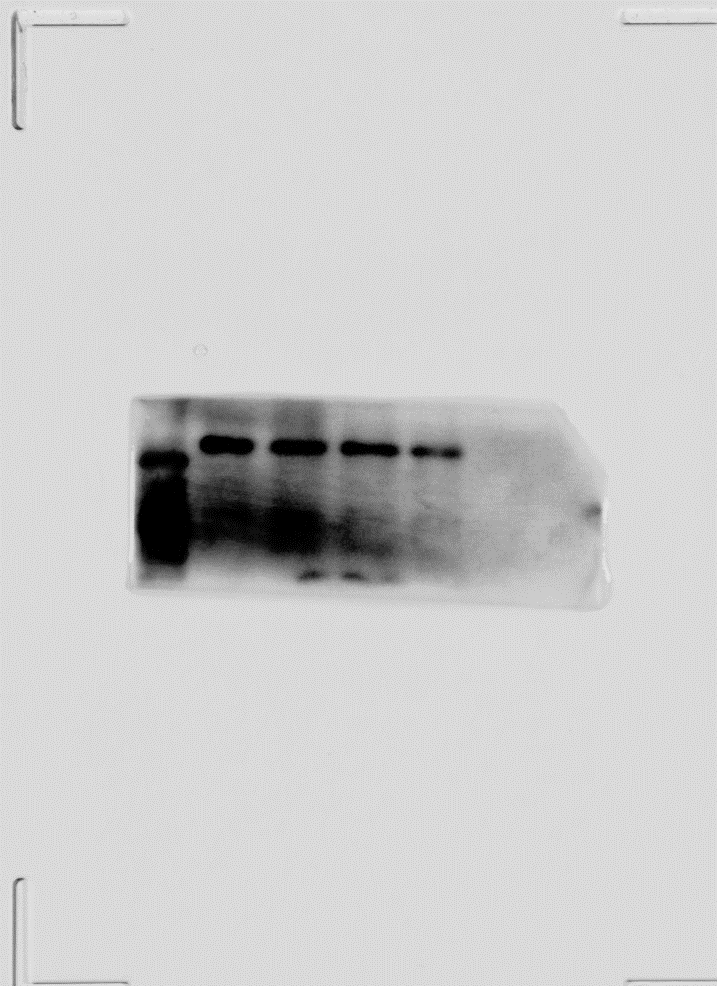
**

**Reference**

1 Demsar, J. *et al.* Orange: Data Mining Toolbox in Python. *J Mach Learn Res* **14**, 2349-2353 (2013).
